# The Impact of Career Plateaus on Job Performance: The Roles of Organizational Justice and Positive Psychological Capital

**DOI:** 10.3390/bs14020144

**Published:** 2024-02-18

**Authors:** Po-Chien Chang, Xinqi Geng, Qihai Cai

**Affiliations:** School of Business, Macau University of Science and Technology, Macau 999078, China

**Keywords:** career plateau, organizational justice, positive psychological capital, job performance

## Abstract

Previous studies suggest that career plateaus have detrimental effects on employees’ satisfaction and performance. Psychological distress generated by career plateaus hinders organizations from achieving the United Nations’ Sustainable Development Goals (UNSDGs) of ‘health and well-being at work’ (SDG-3) and ‘decent work’ (SDG-8). How to mitigate the negative impact of career plateaus becomes the key to enhancing sustainable well-being at work. However, the influencing mechanisms of career plateaus have not been fully discussed, especially regarding employees’ psychological processes. Drawing on the equity theory and the conservation of resource theory, this study examines the influence mechanism of career plateaus on employee job performance via organizational justice, with positive psychological capital moderating the process. Mplus and the Process macro for SPSS are adopted to conduct confirmatory factor analysis and regression analyses. Building on 368 supervisor–employee paired questionnaires with an average of eight employees per supervisor, empirical results indicate that employees who encounter career plateaus reduce their perceived organizational justice to discourage them from performing well in their jobs. Positive psychological capital, however, mitigates the negative effects of career plateaus on perceived organizational justice and the indirect effects of career plateaus on job performance through organizational justice. Theoretically, this study advances our understanding of the influence mechanism of career plateaus on employees’ job performance. Practical implications are also drawn for organizations to alleviate the negative impact of career plateaus to promote sustainable well-being at work.

## 1. Introduction

Under the sluggish market environment resulting from COVID-19, employees are increasingly faced with the practical problems of a single development channel, narrow space for growth, and low mobility. These difficulties and obstacles in the path of career development make the phenomenon of a career plateau more prominent for employees [1]. It is detrimental to the career development of employees to stay in the same position for a long time and engage in repetitive tasks. If employees believe that the organization cannot give them the proper position and treatment or even development space (hierarchical plateau), their work performance will decline. With the increasing division of labor in modern production, employees who have been stuck in a single work position for a long time tend to feel burned out by the familiar and repetitive work content (content plateau) [2]. Hurst (2017) argues that career plateaus lead to inappropriate behaviors and slow performance growth of employees [3].

Previous studies suggest that a career plateau is closely related to a serial status of psychological distress (e.g., stress, burnout, emotional exhaustion, depression), thus jeopardizing employees’ well-being at work [4,5,6,7]. Employee well-being at work has drawn tremendous scholarly attention, especially after the promulgation of the United Nations’ Sustainable Development Goals (UNSDGs) [8,9,10,11]. The UNSDGs consist of 17 Sustainable Development Goals (SDGs) and 169 targets [12]. In particular, career plateaus hinder organizations from achieving the UNSDGs of ‘health and well-being at work’ (SDG-3) and ‘decent work’ (SDG-8). In the context of UNSDGs, how to mitigate the negative impact of career plateaus becomes the key to enhancing sustainable well-being at work. However, the influencing mechanisms of career plateaus have not been fully discussed, especially regarding employees’ psychological processes.

Psychological distress generated by a career plateau is easily attributed to workplace characteristics. Adams (1965) proposed the equity theory, which defines equity as “a type of justice based on merit or contributions” [13]. Equity theory states that individuals are concerned about the fairness of their social relationships and determine fairness by perceiving whether there is a change in the ratio of their input to their reward. A sense of injustice affects an individual’s work attitude and performance; scholars have begun to pay attention to how people construct their group position in a specific situation through a sense of fairness [13,14]. In the context of the workplace, the factors of employee perceived engagement include education, seniority, effort, experience, personality traits, intellectual ability, creativity, loyalty to the organization, and skills. Factors of perceived reward include intrinsic rewards (e.g., a sense of competence and/or purpose), seniority benefits, fringe benefits, status symbols, job security, career advancement, recognition, opportunity for personal development, and participation in important decisions) [15]. Previous studies mainly focused on employees’ perception of fairness through comparison with their colleagues [16] and also found that factors including system, time, and organizational commitment also affect employees’ perception of fairness [17]. In terms of organizational management, unfair systems and allocations first undermine employees’ rights, and then create a sense of unfairness after “comparing others”, a persistent negative effect that leads to employee dissatisfaction and lower performance [18]. Career plateau employees are not satisfied with their current position in the organization, the title they hold, and the job content they are fit for, and when they feel that their abilities are not recognized and their efforts are not reciprocated, they will have a sense of injustice, which will eventually be reflected in their negative work attitudes and slacking behavior. Thus, investigating the impact of career plateaus through the perspective of equity theory has the potential to better reveal the complex influencing mechanism.

At the same time, employees, as the essential human capital of an organization, determine the productivity, innovation, and competitiveness of the organization [19]. Individual characteristics and personal traits moderate the link between a career plateau and perceived fairness, thereby influencing attitudes and behaviors. An essential individual trait in this context is positive psychological capital. According to the conservation of resources theory, employees will increase the input of resources to mitigate the effects of negative emotions caused by resource depletion [20]. When employees face a hierarchical plateau or content plateau, they will pay more attention to and protect their resources. If employees have high positive psychological capital, they are more willing to face difficulties and challenges in their career positively, achieve self-improvement, and thus improve their performance [21]. Reduced opportunities for advancement and unequal distribution of resources lead to stagnation in the organization, which requires employees to have both the ability to respond quickly to job content and the psychological capacity to cope with diverse and resilient career development. Therefore, employees with higher positive psychological capital will take the initiative to reduce the negative impact of career plateaus on their careers through a positive mindset, which is the key to enhancing employee’s sustainable well-being.

By examining the mediation mechanism and exploring the boundary conditions between career plateaus and employees’ job performance, this study makes three notable contributions. First, this research underscores the detrimental nature of a career plateau and its consequential impact on job performance. It provides valuable insights into the application of the equity theory and the conservation of resources theory in the context of social sustainability and the UNSDGs. Second, the study systematically investigates the distinct psychological processes associated with employee job performance in response to a career plateau. This examination enhances our understanding of the nuanced psychological mechanisms involved in career plateaus. Third, this study explores the boundary condition of positive psychological capital, contributing to a deeper comprehension of individual differences in handling career plateaus. In sum, this study aims to advance our understanding of the complexities surrounding career plateaus in the workplace, offering valuable insights for both theoretical development and practical implications in promoting sustainable well-being at work.

## 2. Literature Review and Hypotheses

### 2.1. Career Plateaus and Organizational Justice

From a career management perspective, the phenomenon of a career plateau occurs when employees perceive themselves to be stagnant at a certain point in their careers, resulting in a low likelihood of upward mobility [22]. Employees at a career plateau have poorer performance in terms of in-role behaviors, extra-role behaviors, and work engagement [23]. Career plateaus can cause not only physical and mental discomfort and burnout of employees but even low morale and low efficiency [24]. Employees who are stuck in a career plateau may face two types of stagnation, organizational structure (hierarchical plateau) or job content (content plateau) [7]. When employees feel that their career development is restricted due to an unreasonable organizational structure, it affects their mindset about their future career development [1]. The employees psychologically believe that they cannot get promoted even if they work hard, which leads to slackness and affects their work performance. Employees with job-content stagnation feel that they are not challenged by their work and have no sense of novelty, which reduces their commitment to their work and affects their performance.

According to the equity theory, employees expect to be treated fairly by managers and organizations. If they are treated unfairly, their attitude toward work and performance will be negative [25]. Due to unreasonable organizational structures, employees get stuck in their careers. When they realize that their performance is no longer valued or recognized by the organization, this will affect their perception of distributive justice and cause them to think that their contribution to the organization will get unfair results, thus reducing work efficiency. Employees with job-content stagnation have mastered all the job-related knowledge and skills and can no longer learn new knowledge from their current jobs.

When employees encounter career plateaus, their needs for belonging, meaning, and self-esteem are not met, and they perceive the organization as unfair, which leads to negative behaviors [26]. Therefore, both hierarchical plateaus and content plateaus produce negative effects on employees’ perceived organizational justice:

**Hypothesis** **1.** *Career plateaus are negatively associated with organizational justice.*

### 2.2. Organizational Justice and Work Performance

According to the equity theory, the perception of fairness as the basis for job-related motivation can influence the behavior and affective responses of job performers [13]. In normal workloads, a sense of fairness is beneficial for improving people’s performance [27]. Employees evaluate the exchange relationships with the organizations they are affiliated with in terms of a ratio between effort spent and rewards received at work [13]. Research on compensation shows that when employees discover their advantages through comparison of disposable income between organizations, they generate a sense of fairness through economic exchange, stimulate a comparison favorability impression of the organization, and ultimately increase their desire for increased performance. In other words, when employees perceive that their efforts are rewarded, there is a motivation to reciprocate higher levels of individual performance to the organization [28]. However, as organizations increasingly implement personalized compensation systems, researchers have found that peer comparison often reduces employee perceived fairness. When a company introduces pay-for-performance idiosyncratic deals, the performance level of its peers is immediately negatively affected [29]. A reasonable explanation is that when the difference in rewards received by employees can be rationalized by the difference in employee contributions, employees will gain a sense of fairness, and differentiated treatment can stimulate their desire to improve performance [30]. Therefore, this study establishes the following hypothesis:

**Hypothesis** **2.** *Organizational justice is positively associated with job performance.*

### 2.3. The Mediating Effect of Organizational Justice

A career plateau is a long process, and continuous stagnation leads to employees gradually feeling that the organization no longer supports their career development. Subsequently, employees feel less satisfied with their jobs and plan their careers with a more negative mindset [1]. According to equity theory, employees will gather information through various indirect channels, such as observing the promotion of others or the reasonableness of resource allocation, to judge whether they are in a fair environment, and this informal and emotional judgment often brings more unfairness [17]. When employees are at a career plateau, the unreasonable structure of the organization or the repetitive work content will make them feel unbalanced and believe that the reward is not in line with their contribution and does not meet the psychological fairness condition. Thus, employees are less motivated to complete the work and reduce the time of work or the quality of work to obtain the inner perception of fairness. Therefore, the following hypothesis is proposed:

**Hypothesis** **3.** *Organizational justice mediates the relationship between a career plateau and job performance.*

### 2.4. The Moderating Role of Positive Psychological Capital

In the face of external organizational stress, employees exhibit different behaviors depending on their personality, competence, and effectiveness. Positive psychological capital contains four constructs: self-efficacy, hope, optimism, and resilience [31]. Gardner et al. (1989) confirmed that self-efficacy is significantly related to job performance. Employees with high self-efficacy are more likely to perform well in the workplace and are willing to make efforts to pursue success [32]. Millner (2012) demonstrated that employees with an optimistic attitude can positively view the future development of the work category and as a result, develop a positive working attitude, try to accept new ideas, and strive to make pioneering performance [33]. Larson and Luthans (2006) believe that under goal-oriented motivation, employees with high hopes will start to carry out the course of action to achieve the goal and strive to achieve the goal, driven by hope [34]. Therefore, employees with hopes will work more actively and have more positive behavior outputs. Luthans et al. (2005) believe that employees with resilience can quickly adapt to various situations in the workplace and seek improvement in work performance [35].

The psycho-emotional state of employees is affected by the workplace environment, interpersonal relationships, and work stress. Employees with low psychological capital are more susceptible to emotional sway and further amplify negative effects [36]. Such employees cannot adjust their mindsets at times when they encounter career plateaus; they magnify their losses and blame the organization. Employees’ judgment of organizational justice is influenced by the ratio of gains to losses in their work, and employees’ perception of organizational justice is an important factor for employees to maintain a positive work attitude [37]. Employees with high positive psychological capital will have higher self-efficacy to solve difficulties when facing career plateaus, will face real problems with more optimism, will be objective and hopeful in their evaluation of the organization, and will be more resilient to face challenges in their careers.

Conservation of resources theory suggests that when resource depletion occurs, increasing the input of resources can mitigate the effects of negative emotions [38]. Resources here can be personal psychological characteristics, including self-esteem, working ability, self-efficacy, and a sense of control, which can help individuals resist pressure [39]. When employees feel bored at work and hopeless of promotion, it is a threat to their own resource consumption, and employees will pay more attention to and protect their resources [40]. Therefore, employees in a career plateau perceive the imbalance between resource input and return and thus generate a sense of injustice, which ultimately leads to a reduction in work performance. At the same time, employees with high positive psychological capital will increase their input of personal resources in the face of resource loss in order to protect the resources they deserve. Therefore, employees will invest more positive psychological capital to reduce the loss of resources due to the career plateau. Such employees can actively cope with various difficulties and maintain or even improve their work performance despite their career plateaus [12]. Based on the above derivation, this study proposes that positive psychological capital moderates the mediating effect of perceived organizational justice on career plateaus and job performance. Therefore, this study establishes the following hypothesis:

**Hypothesis** **4.** *Positive psychological capital moderates the relationship between career plateaus and organizational justice, such that this relationship will be weaker when positive psychological capital is high than when it is low.*

Building on the above hypotheses of mediation and moderation, we propose the first-stage moderated mediation hypothesis. Positive psychological capital moderates the relationship between career plateaus and organizational justice, subsequently affecting employees’ job performance. Specifically, for employees with high positive psychological capital, the indirect effect of career plateaus on job performance through organizational justice positive affect is weaker, and stronger for those with low positive psychological capital. Thus,

**Hypothesis** **5.** *Positive psychological capital moderates the mediating relationship of career plateaus and job performance through organizational justice, such that the mediated relationship will be weaker when positive psychological capital is high than when it is low.*

**Figure 1:**
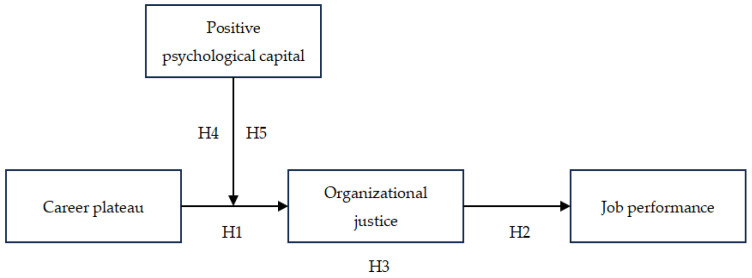
Proposed moderated mediation model.

According to the above-proposed hypotheses, this study proposes the following moderated mediation model (See Figure 1).

## 3. Research Methods

### 3.1. Sample and Procedure

This study adopted a supervisor–employee paired questionnaire survey method. The questionnaires consisted of two parts. The first part was a self-administered questionnaire for employees, covering career plateaus, organizational justice, positive psychological capital, and demographic information. The second part was a supervisor questionnaire, completed by the employee’s direct supervisor, which included the assessment of the employee’s job performance. 

The distribution was based on a ratio of one supervisor to ten employees within each department. A total of 420 employee questionnaires were distributed, along with corresponding supervisor questionnaires given to 42 supervisors coming from 14 organizations. The employee questionnaires were numbered, and supervisors evaluated the job performance of each employee based on the assigned numbers. After removing invalid questionnaires, 368 valid supervisor–employee paired questionnaires were obtained. On average, each supervisor completed evaluations for approximately eight employees, resulting in a valid response rate of 87.6%.

In the 368 valid employee questionnaires, there were 146 males (39.7%), and 222 females (60.3%) in the valid employee questionnaires. Of the total, 159 employees (43.2%) were in the age group of 20 to 29 years old, indicating that the sample for this study was relatively young. In addition, the educational qualifications of the surveyed employees were mainly concentrated in bachelor’s and master’s degrees, 170 (46.2%) and 123 (33.4%), respectively. In terms of working experience, 20.1% of the respondents had between 3 and 5 years of working experience, followed by 95 (25.8%) with 6–10 years of working experience; only 65 (17.7%) had 3 years or less, and 134 (36.4%) had 10 years or more.

### 3.2. Measures

The variables involved in this study were derived from well-established scales. In order to ensure the appropriateness and validity of these measures within the Chinese context, a back-to-back translation procedure was adopted for scales originally formulated in English.

To effectively capture participants’ responses, a five-point Likert-type scale was utilized to measure all the variables under investigation. This scale allowed participants to express their agreement or disagreement, with ratings ranging from 1 (strongly disagree) to 5 (strongly agree).

Career plateaus: We utilized a 12-item, two-dimensional scale developed by Milliman (1992) to assess career plateaus [41]. Sample items were “My opportunities for upward movement are limited in my present organization” (for a hierarchical plateau, with six items) and “I am accountable for the success or failure of my career” (for job-content plateaus, with six items). The Cronbach’s alpha for this scale was 0.97.

Organizational justice: We utilized a 20-item, three-dimensional scale developed by Niehoff and Moorman (1993) to assess organizational justice [42]. Sample items of this scale were “My work schedule is fair” (for distributive justice, with five items), “Job decisions are made by the general manager in an unbiased manner” (for formal procedures, with six items), and “My general manager explains very clearly any decision made about my job” (for interactional justice, with nine items). The Cronbach’s alpha for this scale was 0.97.

Positive psychological capital: We utilized a 12-item, one-dimensional scale developed by Luthans et al. (2008) to assess positive psychological capital [43]. A sample item of this scale was “I feel confident in representing my work area in meetings with management”. The Cronbach’s alpha for this scale was 0.98.

Job performance: We utilized a 10-item, one-dimensional scale developed by Wright et al. (1995) to assess job performance [44]. A sample item was “He/She gets along well with co-workers”. The Cronbach’s alpha for this scale was 0.94.

Control variables: To reduce potential confounding effects, we included gender, age, education, and tenure as control variables in our analyses. According to prior research, demographic characteristics are often considered as determining factors influencing work performance [45,46]. In particular, gender, age, education, and organizational tenure are identified as antecedents of an individual’s behavioral motivation and eventual performance outcomes [47,48,49]. For example, Roberts et al. (2006) found that individuals become more conscientious, extraverted, and less neurotic as their abilities and age increase over time, leading to better performance outcomes [50]. 

## 4. Results

### 4.1. Confirmatory Factor Analysis

The discriminant validity of all the variables adopted in this study was assessed by employing Mplus 8.3 to conduct a series of confirmatory factor analyses (CFA) [51]. The results, presented in Table 1, indicate that the hypothesized four-factor model, consisting of career plateau, organizational justice, positive psychological capital, and job performance (χ^2^ = 3417.71, df = 1371, CFI = 0.91, TLI = 0.90, SRMR = 0.06, RMSEA = 0.05), demonstrated a superior fit to the data compared to alternative one-, two-, and three-factor models. Consequently, these findings supported the idea that our research variables can be considered distinct constructs for further analyses.

### 4.2. Descriptive Statistics and Correlations

Table 2 presents descriptive statistics and correlations for all the research variables. Career plateau was negatively related to job performance (r = −0.38, *p* < 0.001). Additionally, career plateau was negatively related to organizational justice (r = −0.18, *p* < 0.01), and organizational justice was positively related to job performance (r = 0.74, *p* < 0.001). Therefore, the results shown in Table 2 provide preliminary support for Hypothesis 1 and satisfy the preconditions for testing the mediation effect.

### 4.3. Hypothesis Testing

In accordance with Edwards and Lambert’s (2007) path analytic procedures, we employed a specialized SPSS macro developed by Preacher et al. (2007) to examine the mediation and moderated mediation effects [52,53]. Furthermore, we conducted a bootstrapping analysis to evaluate the significance of the conditional indirect effect of the independent variable on the dependent variable across varying values of the moderator.

The findings obtained from the mediation analysis, as presented in Table 3, reveal several significant relationships. Firstly, career plateau exhibited a negative association with job performance (B = −0.42, *p* < 0.001) (see Table 3, Model 1). Secondly, career plateau demonstrated a negative relationship with organizational justice (B = −0.23, *p* < 0.001) (see Table 3, Model 2). Thus, Hypothesis 1 was supported. Lastly, organizational justice exhibited a positive association with job performance (B = 0.76, *p* < 0.001) (see Table 3, Model 3). Thus, Hypothesis 2 was supported. These observed relationships satisfy the three preconditions for testing the mediation effect, as proposed by Baron and Kenny (1986) [54]. Consequently, we proceeded to incorporate both career plateau and organizational justice simultaneously into the regression model. The results indicated that when accounting for organizational justice, the effect of career plateau on job performance (B = −0.18, *p* < 0.001) weakened, while the effect of organizational justice on job performance (B = 0.71, *p* < 0.001) remained significant, indicating partial mediation. To further substantiate the mediation effect, this study employed the bootstrap method to conduct the analysis. As shown in Table 3, at bootstrap = 5000, the 95% confidence interval (95% CI) for the indirect effect is [−0.24, −0.08], signifying that organizational justice acts as a mediator between career plateau and job performance. Thus, Hypothesis 3 was supported by the findings.

To verify the mediated moderation model, we examined the conditional indirect effect of career plateau on job performance through organizational justice. As presented in Table 4, the statistical results show that the conditional indirect effect was smaller at a high level of positive psychological capital (boot indirect effect = −0.07, SE (boot) = 0.03, 95% CI [−0.12, −0.02]) than at a low level of positive psychological capital (boot indirect effect = −0.16, SE (boot) = 0.03, 95% CI [−0.21, −0.10]). These findings provide support for Hypotheses 4 and 5.

**Figure 2:**
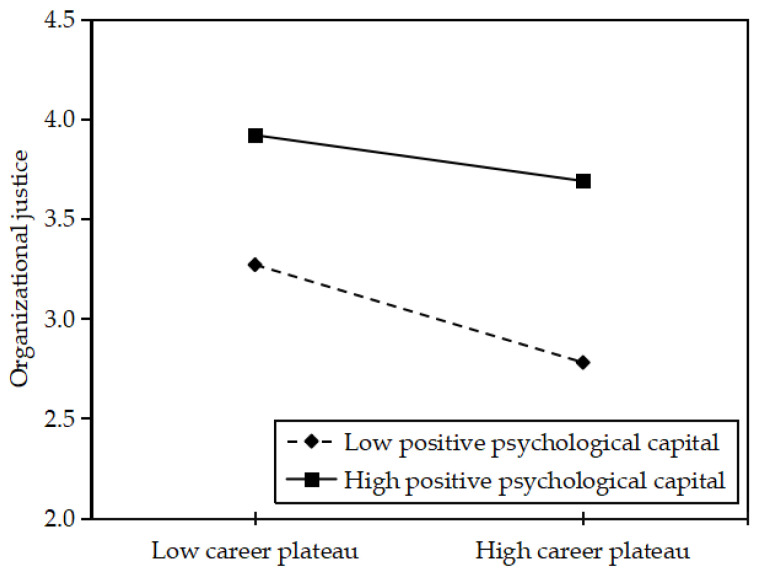
The moderating role of positive psychological capital on the relationship between career plateaus and organizational justice.

To illustrate the interaction effect across different levels of the moderator (mean ± 1 SD), we have depicted these two interactions in Figure 2. As expected, the negative association between career plateaus and organizational justice is weaker when there is more positive psychological capital. 

## 5. Discussion

With the disturbing impact of the COVID-19 pandemic, companies worldwide have had to flatten their organizational structures to cope with the changing environment [55]. The phenomenon of career plateaus is even more prevalent, hindering the achievement of sustainable well-being in the workplace [56]. However, the mechanisms between career plateaus and employees’ behaviors and performance have not been fully discussed, especially regarding employees’ psychological processes. Based on the equity theory and conservation of resources theory, this study investigates the mediating effect of organizational justice on employees’ career plateaus and job performance, and the moderating effect of positive psychological capital. Research results show that employees in career plateaus feel that the input and reward are not proportional and are prone to unfairness, while those employees with higher levels of positive psychological capital can face the difficulties in their career positively and self-regulate to reduce the negative impact of career plateaus on job performance through the sense of organizational justice.

### 5.1. Theoretical Contributions 

The theoretical contributions of this study lie in the following three aspects:

First, the current study enriches the existing literature by revealing the detrimental nature of a career plateau and its consequential impact on job performance. Psychological distress brought on by career plateaus is one of the major obstacles to achieving employees’ sustainable well-being at the workplace. By identifying organizational justice as a mediator and positive psychological capital as a moderator, this study provides valuable insights into the application of the equity theory and the conservation of resources theory in the context of social sustainability and the UNSDGs. 

Second, based on the equity theory, this study reveals the psychological mechanisms through which a career plateau influences job performance by introducing organizational justice as a crucial mediating variable. The existing literature suggests that the impact of career plateaus on work performance is inconclusive. Ference et al. (1977) first proposed that career plateaus are not necessarily associated with poor job performance [22]. Nachbagauer and Riedl (2002) reported similar results [6]. However, most of the empirical examinations provide evidence for a negative association between career plateaus and positive working behavior. For example, Lemire et al. (1999) found a negative correlation between a hierarchical plateau and perceived job performance [57]; Allen et al. (1998) found that the self-evaluation performance of job content plateaued managers was lower than that of non-plateaued colleagues [58]; Hurst et al. (2017) observed a negative correlation between job content, career, and organizational citizenship behavior [3]. This study indicates that a career plateau has negative effects on job performance through perceived organizational justice, adding insights into the existing literature. This finding enhances our understanding of the nuanced psychological mechanisms involved in career plateaus.

Third, this study clarifies that positive psychological capital as a boundary condition mitigates the detrimental effects of career plateaus on organizational justice and subsequent job performance. Prior research suggests that individual differences influence the extent to which employees experience negative outcomes when encountering career plateaus. For example, Milliman (1992) found that a greater desire for promotion strengthens the negative impact of a hierarchical plateau on organizational commitment, turnover intention, and job satisfaction outcomes [43]; Davenport (1993) also reported that the desire for promotion exacerbates the negative impact of a job-content plateau on outcomes [59]. Therefore, the different expectations and efficacy of employees have an impact on the impact of career plateaus, individuals may be able to compensate for plateaus, allowing for their negative effects. This study argues that positive psychological capital serves as a personal resource for coping with a career plateau and plays a pivotal role in career management within the workplace, providing valuable supplementation to the study of career plateaus. In this regard, extrinsic organizational support combined with intrinsic psychological adjustment will be more effective in reducing the negative impact of career plateaus [60]. 

### 5.2. Practical Implications

Two practical implications can be drawn from this study. First, research findings suggest that career plateaus have negative effects on job performance through organizational justice. Therefore, it is necessary for organizations to pay attention to creating a fair organizational climate. For example, organizations should maintain a fair and transparent performance management system to alleviate the negative effects of career plateaus and enhance a sense of fairness among employees. In addition, the establishment of a job rotation system allows employees to work in various positions in the organization and to be exposed to different job content, which can improve their knowledge and skills more comprehensively and reduce burnout caused by being in the same position or repeating the same job for a long time [61]. Job rotation reduces employees’ perceptions of job stagnation, providing them with opportunities to move to parallel positions and increasing their job diversity. Under such circumstances, employees can be motivated to do their jobs more effectively.

Second, our study suggests that positive psychological capital mitigates the negative effects of career plateau on perceived organizational justice and subsequent job performance. Luthans et al. (2007) argue that psychological capital is a psychological resource that is extensible and malleable [31]. Therefore, organizations can help employees who are at a career plateau to rebuild their self-confidence and plan their careers through training and mentoring [62]. Employees with higher levels of positive psychological capital will contribute more to the organization, and the organization should pay attention to the construction of employees’ psychological capital and cultivate talents with high psychological capital.

These proactive approaches have the potential to diminish the detrimental effects of career plateaus on employees’ satisfaction and performance. According to the UNSDG initiative, organizations should accommodate appropriate settings and generate specific conditions to achieve ‘health and well-being at work’ (SDG-3) and ‘decent work’ (SDG-8) [63,64]. Under such circumstances, a ‘sustainable well-being–productivity synergy’ is more likely to be achieved [9].

### 5.3. Limitations and Future Directions

Three limitations of this study need to be mentioned. First, this study employed cross-sectional data and did not track the long-term change in employees’ career plateaus. This limitation may lead to an incomplete understanding of how career plateaus affect employees’ perception of organizational justice over time. Future research adopting a longitudinal study design to track the evolving impact of career plateaus is necessary.

Second, this study did not distinguish between specific occupations, and different occupations may have systematic differences in generating career plateaus due to varying work characteristics. Future research could investigate specific occupations, such as civil servants or nurses, to study the impact of career plateaus on job performance.

Third, the study only examines the moderating role of positive psychological capital, which is an individual-level boundary factor. However, other contextual boundary factors, such as organizational climate, human resource policies, etc., also influence the impact of career plateaus. Therefore, future studies could explore the influence of other contextual boundary factors on the mechanism of career plateaus on job performance through organizational justice, extending our understanding of career plateaus. 

## 6. Conclusions

This study constructs a moderated mediation model that explains the career plateau phenomenon from the perspectives of equity theory and conservation of resources theory. When employees encounter a career plateau, they assess their situation in the organization in a negative direction and compare their gains and losses with a negative mindset, resulting in a sense of unfairness. Subsequently, employees gradually disengage psychologically from the organization and reduce performance, and this negative effect may be reinforced in the long run. However, employees’ levels of positive psychological capital diminish the negative effect of career plateaus. This study provides insights for future studies and practices aimed at better understanding employees’ sustainable well-being at work in the context of career plateaus, with the goal of promoting the Sustainable Development Goals.

## Figures and Tables

**Table 1:** The results of confirmatory factor analyses.

Measurement Model	χ^2^	df	Δχ^2^	CFI	TLI	SRMR	RMSEA
The hypothesized 4-factor model	3417.71	1371		0.91	0.90	0.06	0.05
3-factor model (combining CP and JP)	5808.81	1374	2391.10	0.80	0.79	0.09	0.21
2-factor model (combining CP, OJ, and JP)	8618.78	1376	2809.97	0.67	0.65	0.12	0.16
1-factor model (combining PPC, CP, OJ, and JP)	13,056.69	1377	4437.91	0.46	0.44	0.15	0.19

Note: *N* = 368; CFI = comparative fit index; TLI = Tucker–Lewis index; SRMR = standardized root mean square residual; RMSEA = root mean square of approximation; CP = career plateau, OJ = organizational justice, PPC = positive psychological capital, JP = job performance.

**Table 2:** Means, standard deviations, correlations.

Variables	Mean	SD	1	2	3	4	5	6	7
1. Gender	0.40	0.49							
2. Age	1.87	0.86	0.21 ***						
3. Education	2.43	0.81	0.13 *	0.13 *					
4. Seniority	2.70	1.16	0.24 ***	0.76 ***	−0.09				
5. Career plateau	2.71	0.99	0.06	0.25 ***	0.22 ***	0.06			
6. Organizational justice	3.42	0.71	−0.16 **	0.07	0.11 *	0.09	−0.18 **		
7. Positive psychological capital	3.64	1.19	−0.08	0.04	−0.03	0.11 *	0.03	0.53 ***	
8. Job performance	3.88	0.64	−0.09	0.04	−0.03	0.08	−0.38 ***	0.74 ***	0.62 ***

Note: *N* = 368; SD: standard deviations; * *p* < 0.05, ** *p* < 0.01, *** *p* < 0.001.

**Table 3:** Regression results for direct effect model and mediation model.

Variables	Model 1 X→Y	Model 2 X→M	Model 3 M→Y	Model 4 X→M→Y
Gender	−0.11 *	−0.21	0.05	0.04
Age	0.12	0.03	−0.01	0.10
Education	0.07	0.19	−0.12	−0.07
Tenure	0.05	0.15	−0.01	−0.06
Career plateau	−0.42 ***	−0.23 ***		−0.18 ***
Organizational justice			0.76 ***	0.71 ***
R^2^	0.18	0.11	0.57	0.54
F	15.58 ***	8.97 ***	94.61 ***	125.80 ***
Bootstrap results for indirect effect				
	M	SE	LLCI	ULCI
Effect	−0.16	0.04	−0.24	−0.08

Note: *N* = 368; * p < 0.05, *** p < 0.001; unstandardized regression coefficients are reported; bootstrap sample size 5000; LL = lower limit; UL = upper limit; CI = confidence interval.

**Table 4:** Regression results for conditional indirect effect.

Predictor	Organizational Justice			
	B		SE	
Moderation model				
Gender	−0.14 **		0.06	
Age	0.12		0.06	
Education	0.19 ***		0.04	
Tenure	0.01		0.04	
Career plateau	−0.25 ***		0.03	
Positive psychological capital	0.55 ***		0.03	
Career plateau × Positive psychological capital	0.12 **		0.03	
Moderated mediation model				
	Effect	BootSE	BootLLCI	BootULCI
Low positive psychological capital (Mean − 1 SD)	−0.16	0.03	−0.21	−0.10
Positive psychological capital (Mean)	−0.11	0.02	−0.16	−0.07
High positive psychological capital (Mean + 1 SD)	−0.07	0.03	−0.12	−0.02

Note: *N* = 368; ** *p* < 0.01, *** *p* < 0.001; bootstrap sample size 5000; LL = lower limit; UL = upper limit; CI = confidence interval.

## Data Availability

The data shown in this research are available on reasonable request from the corresponding author.
